# Characterization of the pollen beetle, *Brassicogethes aeneus*, dispersal from woodlands to winter oilseed rape fields

**DOI:** 10.1371/journal.pone.0183878

**Published:** 2017-08-25

**Authors:** Amandine Suzanne Juhel, Corentin Mario Barbu, Pierre Franck, Jean Roger-Estrade, Arnaud Butier, Mathieu Bazot, Muriel Valantin-Morison

**Affiliations:** 1 UMR Agronomie, INRA, AgroParisTech, Université Paris-Saclay, Thiverval-Grignon, France; 2 INRA, UR1115 Plantes et Systèmes de culture Horticoles, Avignon cedex, France; Northwest A&F University, CHINA

## Abstract

Many crop pests rely on resources out of crop fields; understanding how they colonize the fields is an important factor to develop integrated pest management. In particular, the time of crop colonization and damage severity might be determined by pest movements between fields and non-crop areas. Notably, the pollen beetle, *Brassicogethes aeneus*, previously named *Meligethes aeneus*, one of the most important pests of winter oilseed rape, overwinters in woodlands. As a result, its abundance increases in oilseed rape fields near wooded areas. Here, we assessed the spatio-temporal patterns of the dispersal from woodlands to oilseed rape fields in diversified landscapes of a same region. We observed on four dates the abundance of pollen beetles in 24 fields spread in the Eure department, France. We modeled the abundance as a result of the dispersal from the neighboring woodlands. We compared the modalities of dispersal corresponding to different hypotheses on the dispersal origin, kernel shape and sources of variability. Within oilseed rape the distance to the edges of woodlands is not the main determinant of pollen beetle abundance. On the contrary, the variability of the abundance between fields is largely explained by the dispersal from neighboring woodlands but there is considerable variability between dates, sites and, to a lesser extent, between fields. The two dispersal kernels received similar support from the data and lead to similar conclusions. The mean dispersal distance is 1.2 km but seems to increase from a few hundred meters the first week to more than two kilometers the fourth, allowing the pollen beetles to reach more distant OSR fields. These results suggest that early varieties away from woodlands and late varieties close to the woodlands may limit attacks at the time when oilseed rape is the most sensitive.

## Introduction

Understanding pest dispersal is crucial for the design of integrated pest management strategies, particularly when non-crop areas are potential sources of pests that recolonize surrounding fields [1]. The pollen beetle *Brassicogethes aeneus* (Fabricius, 1775) (formerly *Meligethes aeneus*) (Coleoptera: Nitidulidae) is one of the most important insect pests in winter oilseed rape (OSR; *Brassica napus L*.) in Europe [2]. In many countries, OSR receives a large fraction of the pesticides used on grain crops [3]. As a result, insecticide resistance is widespread among OSR pests; for example, pollen beetles in Europe became generally resistant to pyrethroids [4], [5]. Alternative integrated pest management strategies for OSR crops have been actively sought for in the last decade, such as repellents [6], traps [7] or resistant cultivars [8]. At the field scale, methods such as trap crops have been suggested to limit the damage to the crop [7]. At the landscape scale, it has been suggested to improve the management of semi-natural habitat surrounding crops [9]. The life cycles of the pests or of their natural enemies often involve semi-natural habitats providing overwintering or feeding sites [10]. The pollen beetle eggs hatch in OSR buds, and the larvae develop within buds and drop to the ground to pupate. The new generation of pollen beetle emerges a few weeks later, in the early summer, and seeks overwintering sites in woodlands [11], [12]. In the early spring (March-April), the adults migrate from the woodlands to OSR fields to feed on pollen and oviposit [2]. If they arrive before flowering, they destroy the buds to feed and can inflict severe yield losses [13].

In such a life cycle, dispersal capacities play a crucial role, in particular allowing movements from woodlands to fields and from fields to woodlands [14]. Beyond studying correlations between pollen beetles abundance data and landscape elements [12], [15], [16] characterizing the dispersal could improve the prediction and management of the pollen beetle. As the timing of their arrival in the crop is decisive for the damage, understanding the temporal dynamic of their dispersal would further help the design of management strategies. Finally, considering dispersal both within and between crops would help design attraction based management strategies such as trap crops.

The dispersal distance has been studied by marking insects with radioactive elements [17], [18]. A first study found the pollen beetles to disperse in the spring (March) up to ten kilometers within two days; though ten kilometers was also the maximal distance covered in twelve days [17]. For the summer dispersal (July), a mean distance per days of 1 to 3 km was observed [18]. Dispersal distance can also be estimated fitting dispersal kernels to abundance data, which potentially yields very different estimates [19]. Moreover, pollen beetles flight could be influenced by temperatures [16]; the height of the flight had also been studied [6]. Within a field pollen beetles are more abundant at the edges but little is known on their dispersal in this spatial scale [2], [20], [21].

Here we investigated the mean dispersal distance covered by adult pollen beetles from woodland to OSR fields during spring. We sampled adults in 24 OSR fields over a broad landscape and fit alternative models to these data to characterize the dispersal. Specifically we investigated whether the average dispersal distance changes with time or with the complexity of the landscape. We also tested if the specific location of the points in the fields was relevant or if considering all sampling in a field to be at the same median place was more adequate, suggesting a different type of dispersal within OSR fields. Finally, we investigated if the abundance in the field was best explained by considering the whole area of large woodlands as a source of pollen beetle or by considering only the first 100 m within the woodland from their edges as a source of pollen beetle.

## Material and methods

### Study site

**Fig 1:**
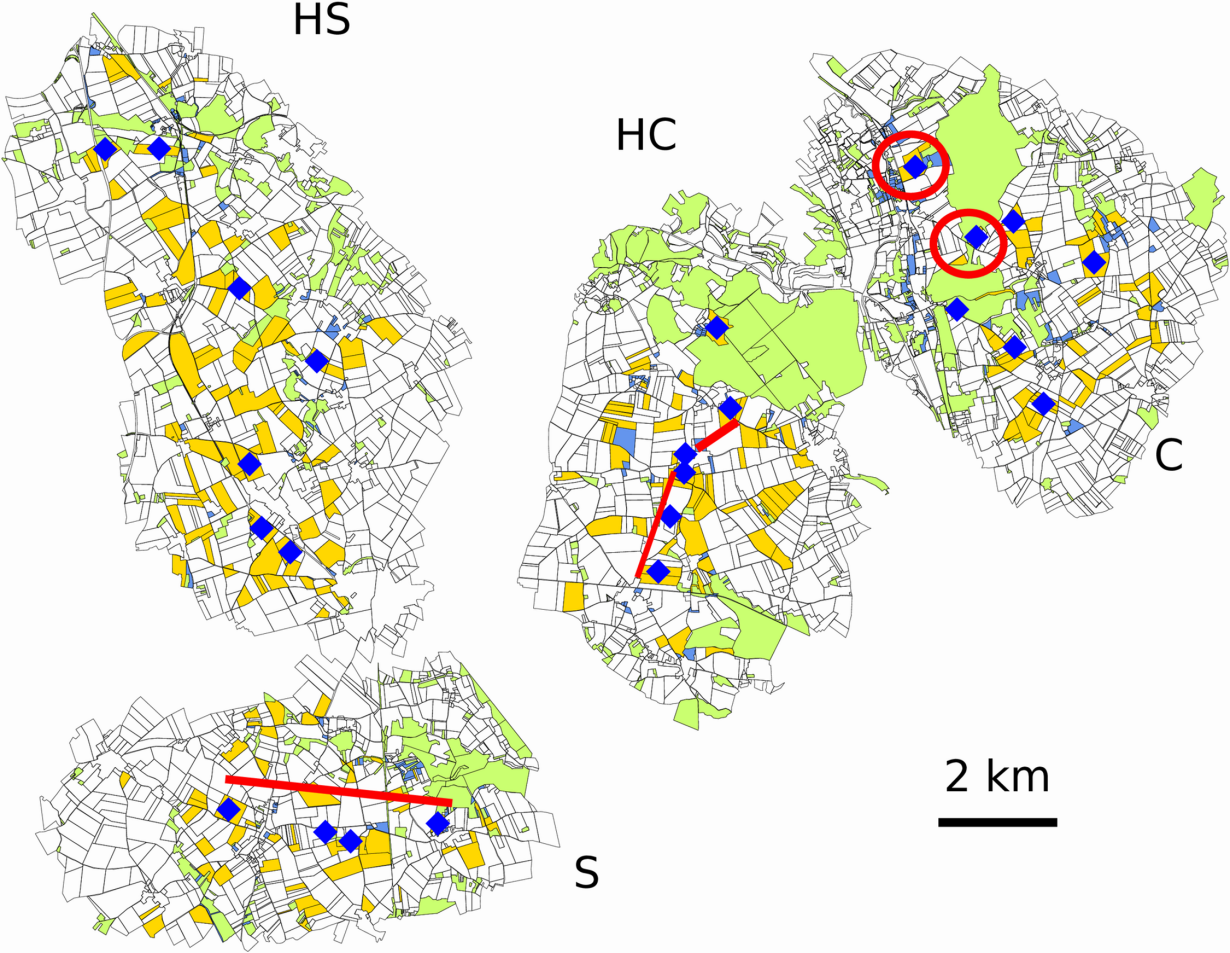
Map of the four studied sites (Normandy region, France), describing land uses within two kilometers radius buffer around the barycenter of the sampled OSR fields in 2015.Green: woodlands. Blue: grasslands. Yellow: OSR crops. Diamond: sampled OSR fields. Red lines: between fields transect lines. Red circles single out fields where we followed within-field transects from woodlands over 200 m. We distinguish four sites with differing complexities: Highly Simple: HS (7 fields), Simple: S (4 fields), Complex: C (7 fields), Highly Complex: HC (5 fields). Complexity here is a gradient of woodland surfaces in each sites, simple is a site with few woodlands.

The study was carried out in 2015, in four agricultural landscapes from the Normandy region in north western France bounded at North West at 49°00'31.9"N 1°10'23.0"E, at South West at 48°51'47.8"N 1°12'51.5"E, at 48°56'56.5"N 1°30'15.6"E for South East and at 49°00'08.6"N 1°27'42.6"E for North East (Fig 1).

This area has a sub-oceanic temperate climate, with mild summers (mean monthly temperature from May to July in 2015, 20.9 [10.7–23.8] °C; monthly mean precipitation: 53.2 mm) and cool late winters (mean monthly temperature for January and February 2015: 4.1 [1.2–7.1] °C; mean monthly precipitation: 45.9 mm). The semi-natural habitat consists in large woodland areas and small woodlots, hedgerows and grasslands; all of which have been related to the life cycle of the pollen beetles [2]. The OSR is cultivated in relatively high proportion of the cultivated fields (Fig 1). We defined four sites of about four by six kilometers with a growing proportion of woodlands. Note that in addition to the growing proportion of woodlands, the first two were on a plateau with little to no hedgerows, while the two others were centered on valleys with a hedged landscape (as qualitatively assessed by visual inspection of the sites) and also with more grasslands. This growing landscape complexity prompted us to label these landscapes by their complexity as Highly Simple: HS, Simple: S, Complex: C, Highly Complex: HC (Fig 1).

### Insects sampling

We selected 24 OSR fields distributed over the four sites (four to seven fields per site, Fig 1). Nine fields, among the 24 selected fields, were chosen aligned along three transects in two sites (Fig 1) from important woodlands in the area (> 1 km^2^), allowing visualization of the gradient of abundance when getting further from these woodlands. In each of the 24 selected fields we sampled five aligned points approximately separated by 50 m. These transects moved from the edge of the field toward the middle of the field perpendicularly to its edge. In two fields at immediate proximity of a major woodland (red circles Fig 1), in the field, we sampled five additional points allowing to visualize a potential gradient of abundance within a field.

At each sampling point, we sampled adult pollen beetle by beating ten plants inside plastic bags. These ten plants were chosen at 1 m from each other to avoid pollen beetles falling from crops while beating nearby. We repeated the sampling four times, one week apart between March 25^th^, 2015 and April 16^th^, 2015. The OSR shifted during this period from bud developments stage (Growth Stage GS 50) to the end of the flowering stage (GS 65). Among the 3657 pollen beetles captured, 230 individuals equally distributed among the four study sites were identified by their meta-femur [22]. Most pollen beetle were identified as *B*. *aeneus* (99%). Only two specimens were identified as *B*. *viridescens* (Fabricius, 1787). Consequently, we did not differentiate the two species in the following analysis of the abundance. For all our analyses, we defined the abundance of pollen beetles as the number of pollen beetles for ten OSR plants, i.e. the number of pollen beetles caught at a given sampling point and date.

### Mapping and geomatics

We mapped forests and field delimitations respectively within five or two kilometers from sampled fields using aerial photographs (GoogleMyMaps pixel size: 0.5 m, 2017 TerraMetrics). To help discussion of the results, we also mapped grasslands and OSR fields within two kilometers of the selected OSR fields (Fig 1). Hedgerows were not mapped as they were more difficult to identify from aerial photographs. To account for wooded areas in the models, we discretized the woodlands on a grid of 100 m resolution (pixel of 100 m*100 m) and affected to each pixel the wooded area it covered. Previous studies did not find differences in emergence abundance after overwintering within the first 100 m within the woodland from their edges than deeper in the woodland [11]. To assess if more central parts of the woodland are less important hibernating sites than the periphery, we here differentiate the 100 m wide periphery from the more central parts of the woodlands.

We calculated woodland areas and distances of each sampling point to woodlands using the R package rgeos [23] using Universal Transverse Mercator (UTM) projection system. All geomatics treatment and statistical analyses were performed using R software (R Core Team 2016).

### Statistical analysis

We modeled the abundance (total number) of pollen beetle at each sampled point as a random variable in a Bayesian perspective. Abundance data are commonly described using a Poisson distribution; here, as the data tended to be over-dispersed we assumed the abundance to follow a negative binomial distribution of mean *μ* varying by date *t* and sampling point *i*. The size parameter for this distribution, strictly positive, was fitted using a flat prior on the log scale.

#### Modeling the mean abundance of pollen beetle

We modeled the expected abundance *μ* observed at a given time as the product of the intensity *γ*_*e*_ of pollen beetle arriving at the sampling point and a categorical size factor *γ*_*g*_:

$$\mu =\gamma_{e}\cdot \gamma_{g}$$ (1)

The size factor *γ*_*g*_ was a catch-all categorical parameter allowing to model different sources of variability. The visibility of the pollen beetle in the crop is highly dependent on the weather [13], in addition the attractiveness of the crop for the pollen beetle varies with time [24], so each date is considered separately in γ_*g*_. We also considered the possibility to have a specific category per location (at field or site level). Finally, we considered the possibility that a different category should be fitted for each location-date couple. For this categorical size factor γ_*g*_ we used as a non-informative prior a Cauchy distribution with center 0 and scale 2.5 [25].

The pollen beetle arriving at a sampling point *i* was the sum of the pollen beetle arriving at date *t* from each surrounding unit of woodland *w* of area *A*_*w*_ within 5 km. The expected number of pollen beetle *μ*(t,i) observed in field *i* becomes:

$$\mu (t,i)=\gamma_{g}\sum_{w}\gamma_{d}(i,w,t)\cdot A_{w}$$ (2)

Where γ_*d*_ (i,w,t) was a dispersal kernel from the unit of woodland *w* to a field *i* at date *t*. This representation does not imply that the flight is direct from the source to the destination, on the contrary, different kernels of dispersal can be interpreted as different types of path, with more or less relay habitatsand more or less random or directed movement to the destination.

#### Dispersal kernels

We tested two commonly used dispersal kernels: the Gaussian kernel and the Exponential kernel [26]. These two contrasted kernels are chosen to make sure the results are not too dependent on the shape of the kernel. These kernels were considered to be a function of the date *t*:

$$\gamma_{d}(i,w,t)=\frac{1}{K\pi {\delta (t)}_{}²}\exp\left(-{\left(\frac{d_{iw}}{\delta (t)}\right)}^{n}\right)$$ (3)

Where *K* and *n* varied with the dispersal kernels (Gaussian kernel: *K* = 1 and *n* = 2, exponential kernel: *K* = 2 and *n* = 1). *d*_*iw*_ was the distance from the woodland unit to the sampled point. *δ*(t) is a scale parameter, homogenous to a distance and a function of time. To test if the dispersal varies with time, we set that the abundance decreases with the distance (1/ *δ*(t)) and changes linearly with time by a factor *β*_*dt*_ each week from an intercept *β*_*d*_ at the first date (*t* = 1) as follow:

$$\delta (t)={1/(\beta }_{d}+\beta_{dt}(t-1))$$ (4)

For the dispersion parameters *β*_*dt*_ and *β*_*d*_ we applied a flat prior on their definition domain. Their definition domain corresponds to a positive distance of dispersion: *β*_*d*_*>0* and *∀ t ∈ {1*,*2*,*3*,*4} β*_*d*_ *+ β*_*dt*_ (t-1) *> 0*.

For an exponential kernel, the mean dispersal distance D_mean_ is given by:

$$Dmean\;(t)=\frac{2}{\left(\beta_{d}+\beta_{dt}(t-1)\right)}$$ (5)

For a Gaussian kernel, D_mean_ is given by:

$$Dmean\;(t)=\frac{\surd \pi }{2\cdot (\beta_{d}+\beta_{dt}(t-1))}$$ (6)

#### Fit and comparison of models

We compared the fit of the models using the total area of the woodlands or just the 100 m peripheral area to test if pollen beetle can be considered to only emerge from the 100 m peripheral area. To evaluate the effect of distances from woodlands within fields, we compared a model considering the actual coordinates of the five points in a field (split) and a model considering the five samples to be at the barycenter of the points (merged). In the second model the position information within the fields is hidden, preventing the fit of a potential gradient within fields. Comparing the two models allows then to say if considering a gradient within the field improves the fit and hence if the gradient from woods levels off or not within fields. We also considered a model without geographical coordinates (“none”) to assess the relevance of accounting for the proximity of woodlands. Finally, to test the effect of landscape complexity on the dispersal, we tested if fitting the parameters β_d_ (factor determining the average dispersal distance from woodlands to OSR) and β_dt_ (factor modifying the average dispersal distance with time from woodlands to OSR with time) separately for the four sites that had different complexities improved the adjustment to the data. Testing different possible categorical factors to describe the location and date allowed to check the robustness of the results to the above tests. Combining these modalities (Table 1) led to test 161 models.

**Table 1:** Parameters and modalities used to test the 161 models.

Parameter	Modalities	Description and hypothesis	Definition domain
***β***_***d***_	***-***	**Dispersal distance factor**	]0;+ ∞[
***β***_***dt***_	***-***	**Modification of the dispersal distance factor by time**	*β*_*d*_ *+ β*_*dt*_ (t-1) *> 0*
**Size factor**		**Date and location categorical effect**	
	Date	Effect per date of sampling	]-∞,+∞[
	Field	Effect per field	]-∞,+∞[
	Site	Effect per sampling site	]-∞,+∞[
	Field + date	Separate field and sampling date effects	]-∞,+∞[
	Site + date	Separate site and sampling date effects	]-∞,+∞[
	Field: date	An effect per field-date couple	]-∞,+∞[
	Site: date	An effect per field-date couple	]-∞,+∞[
	"1"	Same factor for all sampling points and dates	]-∞,+∞[
**Origin**		**Part of woods considered as a source of *B*.* aeneus***	
	Area	Total woodland area	-
	Edge	Edges of woodland area (first 100 m within woods)	-
**Field point structure**		**Coordinates of the sampling points used in the model**	
	Split	Actual coordinates of the five points in a field	-
	Merged	The barycenter of all the points sampled in the field	-
	None	Coordinates not accounted for (no distance effect)	-
**Kernel**		**Shape of the dispersal kernel**	
	Exponential	Exponential decrease from woodlands (oriented dispersal)	-
	Gaussian	Normal decrease from woodlands (random dispersal)	-
**Site dependent dispersal**		***β***_***dt***_ **and *β***_***d***_ **fitted separately in each site?**	
	True	Dispersal varies by site (landscape complexity)	-
	False	Same dispersal in all sites (landscape complexity)	-

Each of these models was adjusted using a Monte Carlo Markov chains (MCMC). Each MCMC was run until the Geweke diagnostic [27] and the Raftery and Lewis diagnostic [28] were satisfied as implemented in the YAMH, R package [29].

The confidence intervals for the estimates correspond to the quantiles 2.5% and 97.5% of the values sampled in the Markov chains. Hereafter the parameter values we present correspond to the set maximizing the likelihood in the Markov chain unless the likelihood corresponding to the median value in the Markov chain for each parameter provides a higher likelihood. We then ranked all the models by their AICs [30].

### Relating pollen beetle abundance to woodland areas in buffers

In a similar way to previously published work [12], [14], [31], we also modeled the pollen beetle count data as a function of woodland areas in buffers around the sampling points:

$$\mu (t,i)=\gamma_{g}\;A_{BW}$$

With A_BW_ the area of woodlands in a given buffer (disk) around the sampling point. We used a glm assuming a negative binomial distribution of the pollen beetle count data, with the canonical log link function. The above becoming:

$$log(\mu (t,i))=\beta_{G}+{log(A}_{BW})$$

Where β_G_ corresponds to the parameters for the categorical factors G factors for time and localization as described for the kernel based models. We fitted the buffer radius by selecting the model with the best likelihood for models with radii ranging from 0.2 to 5 km by increments of 200 m.

## Results

The mapping of the four sites allowed to rank the four sites from highly simple (HS) to highly complex (HC) (Table 2).

**Table 2:** Landscape characteristics of the four study sites in 2009 (within two kilometers of sampled fields). Woodland edges are the first 100 m within the woodlands from its edge.

Site name	Area size (km^2^)	Woodland core	Woodland edges	Grassland	OSR crops	Other crops
Highly Simple (HS)	56.9	7.4%	6.8%	0.8%	14.8%	66.7%
Simple (S)	31.5	13.9%	9.9%	0.7%	8.4%	65.3%
Complex (C)	47.1	18.2%	10.9%	3.0%	8.4%	58.1%
Highly Complex (HC)	35.8	22.7%	10.4%	1.2%	9.9%	54.4%

Area siza (km^2^), proportion (%) of woodlands edges and core, grasslands, OSR and other crops in each site.

Abundance of pollen beetle was low but compared to other observations in Europe [15] but highly variable between sites, fields and even within fields (Table 3). The average pollen beetle abundance was higher in more complex sites and reached its peak on April 8^th^ (t_3_) (Table 3).

**Table 3:** Observed pollen beetles mean abundance [±SE] (number for ten plants) by site and date.

Site	t_1_: March 25^th^	t_2_:April 1^st^	t_3_:April 8^th^	t_4_: April 15^th^	All
HS	2.4 (±5.4)	0.3 (±0.8)	3.9 (±4.8)	1.6 (±1.8)	2.6 (±3.9)
S	1.3 (±3.7)	0.4 (±0.9)	6.4 (±6.4)	2.9 (±2.6)	2.7 (±3.3)
C	2.5 (±3.7)	2.5 (±2.9)	33.8 (±26.2)	12.6 (±12.8)	13.2 (±17.6)
HC	1.3 (±2.2)	0.5 (±0.9)	16.1 (±21.0)	7.2 (±7.8)	6.5 (±11.7)
All	3.8 (±4.3)	0.9 (±1.5)	15.2 (±20.5)	6.7 (±9.1)	

Mean values (± SD).

### Visible influence of the distance from the main woods at the kilometer-scale

**Fig 2:**
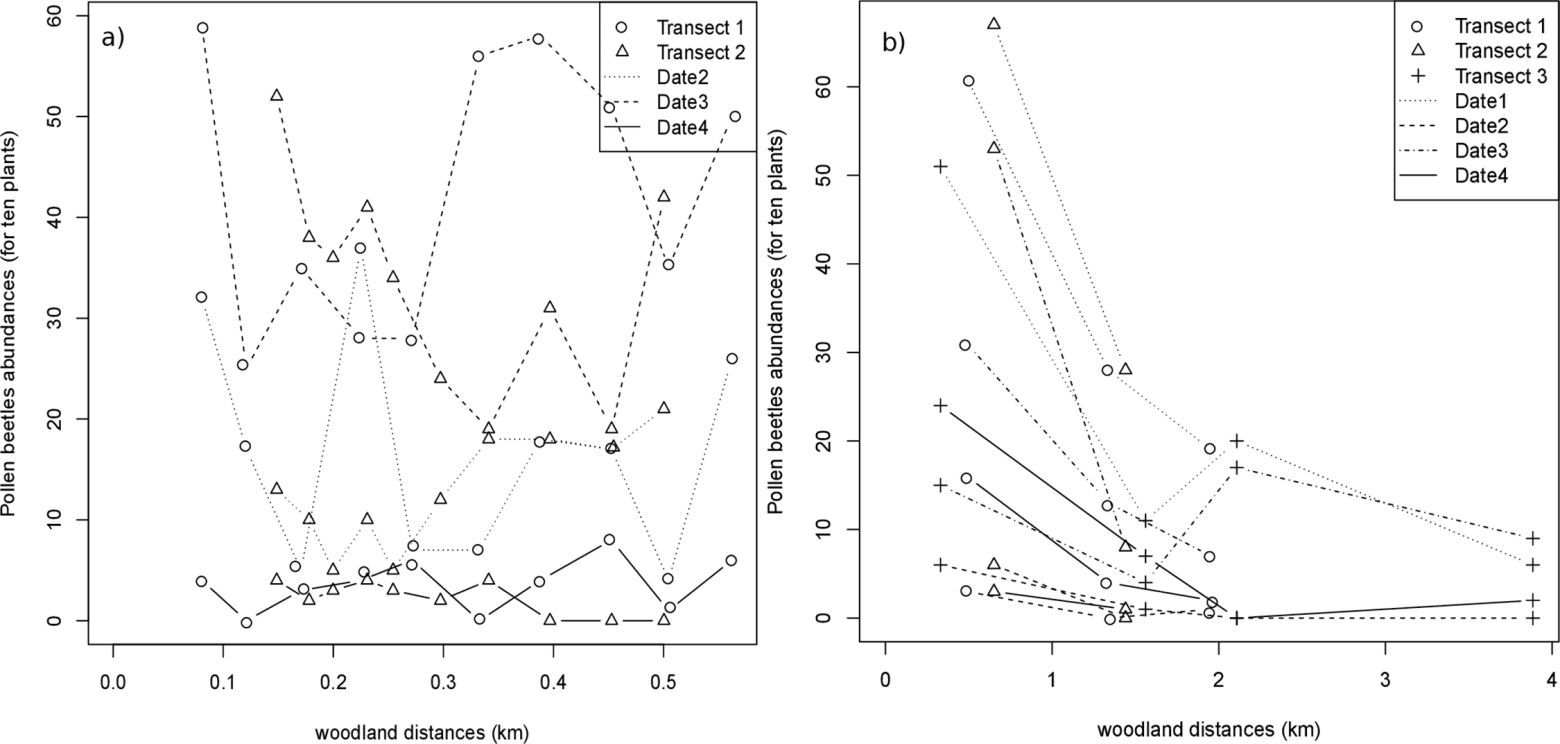
Variation of pollen beetle abundance along within-field (A) and between-field (B) transects as a function of distance (km) to woodland edges. (A) Number of pollen beetles per sampling point within two different OSR fields (fields in complex site, C). (B) Mean number of pollen beetles per field in three transects (in zones S, for simple landscape, and HC, for highly complex landscape). Abundance were measured on 10 OSR plants at each sampling point during three (A) or four (B) consecutive weeks (dates 1 to 4). Trans: Transect.

The abundance easily reaches 5-fold variations in a given field at a given date. Within field abundance were highly variable and in a case the highest abundance in a field was reached in the middle of the field (Fig 2A).On the contrary, the abundance of pollen beetles merged by field show a clear and quick decrease of pollen beetles abundance with the distance from woodlands over a few kilometers (Fig 2B). The impact of the date is also major with very few pollen beetle captured on the date 2.

### Significant effect of the date, the field and dispersal from woods

Comparing the AIC of the 161 models, allowed us to identify the dispersal modalities best explaining the observed patterns (Table 4), all models are presented in S1 Table and Data are available in the S2 Table. The best model (Table 4, N°1) accounted for the effect of fields and the effect of the date separately, it kept the spatial structure of the points within a field (split), used a Gaussian kernel and the whole area of the woods was considered as a source of pollen beetle. Finally, the dispersal parameters were fitted separately for each site (Table 4).

**Table 4:** Best models obtained out of the 161 models estimating pollen beetles dispersal from woodlands to OSR, constraining in turn each modality (categorical factor type, origin, Field point structure, kernel and site dependant dispersal) leaving other modalities and parameters free, with or without a field effect. Bold: parameter constrained, allowing a field effect. Underlined: parameter constrained, not allowing a field effect.

N°	Categorical factor type	Origin	Same field point structure	Kernel	Site dependent dispersal	AIC	Δ AIC	Mean β_dt_	Mean D_mean_ (km)
1	**Date+field**	**area**	**split**	**Gaus.**	**TRUE**	2419.8	0	0.22 [0.07,0.38]	3.4 [1.6,5]
2	Date+field	area	split	**exp.**	TRUE	2424.0	4.2	0.57 [0.18,1.07]	4.2 [2.8,6.5]
3	Date+field	**edge**	split	Gaus.	TRUE	2427.7	7.9	0.2 [0.05,0.38]	3.1 [2.4,4.1]
4	**Date:field**	**NA**	**none**	NA	NA	2433.7	13.9	NA	NA
5	Date+field	area	**merged**	exp.	TRUE	2435.4	15.6	0.52 [-0.05,1.4]	4.1 [3.0,6.6]
6	Date:field	area	merged	Gaus.	**FALSE**	2440.1	20.3	0.03 [-0.08,0.16]	2.2 [1.5,3.0]
7	**Date:site**	edge	split	Gaus.	TRUE	2453.3	33.4	0.26 [0.1,0.42]	1.2 [1.0,2.6]
8	Date:site	edge	merged	Gaus.	TRUE	2454.6	34.8	0.16 [0.04,0.29]	1.2 [1.0,1.7]
9	Date:site	edge	merged	Gaus.	FALSE	2454.9	35.1	0.07 [-0.03,0.18]	1.1 [1.0,1.3]
10	Date:site	edge	split	exp.	TRUE	2457.8	38.0	0.61 [0.18,1.06]	1.3 [1,3.1]
11	Date:site	area	split	exp.	TRUE	2466.2	46.4	0.54 [0.14,0.95]	1.8 [1.3,3.0]
12	**Date+site**	edge	merged	Gaus.	TRUE	2474.0	54.1	0.03 [-0.08,0.21]	2.4 [1.5,2.8]
13	**Date**	edge	merged	Gaus.	FALSE	2568.3	148.4	0.09 [-0.02,0.18]	1.1 [0.9,1.3]
14	**"1"**	area	split	exp.	TRUE	2737.4	317.6	1.48 [0.68,2.29]	2.6 [1.4,5.6]
15	**field**	edge	split	Gaus.	TRUE	2770.4	350.5	0.19 [0.03,0.51]	4.1 [2.0,6.2]
16	**site**	area	none	Gaus.	FALSE	2934.9	515.1	NA	NA

Factor type: type of factor. Origin: whole wood area or only 100 m wide area bordering woodland area. Same field point structure: split = with real coordinates of points. Same field point structure = all points of a field with coordinates of barycenter, none = no coordinates. Kernel = Exponential (exp.) or Gaussian (Gaus.) dispersal kernels. Site dependent dispersal: with or without interaction between site and distance. Δ AIC: difference of AIC with first model. Mean β_dt_: mean effect of time on the dispersal distance. Mean D_mean_: mean dispersal distance from woodlands to OSR.

Removing the date size effect had the strongest impact on the AIC: the models without date effect constitute the top were the worst, several hundred points of AIC behind the best. Including a location effect, at least at site level was also important as it allowed to get within a hundred AIC points of the best model. Allowing an interaction between site and date effect further improved the description of the data by 20 points of AIC. Using date and field effect separately had a clear impact with a difference of 5 points of AIC.

Constraining the dispersal still had a strong impact (Δ AIC > 10 points) whether the dispersal was forced to be homogeneous between sites, the points within a field were merged or no effect of the distance to the woodlands were considered. Only considering the 100 m periphery of the woodlands as sources had a limited impact on the fit (AIC variation of seven points and similar estimates). Finally, the best model retained a Gaussian kernel but imposing an exponential kernel had a limited impact on the fit (AIC variation of four points) and yield similar mean dispersal distances and impact of time on the dispersal (Table 4).

### The field effect obfuscates the dispersal from woodlands in complex sites

Measuring the importance of the woodlands by differentiating the best model (Table 4, N°1) with the best model without woodlands (Table 4, N°4) can minimize the effect of woodlands as the variability otherwise explained by the woods is transferred to the field effect. Using as reference the best model without field effect (Table 4, N°7), the Δ AIC, removing the effect of woof (S1 Table, N°87), attained 180 confirming the importance of the woodlands.

Using as a reference the best model without field effect also changed the relevance of some dispersal modalities (Table 4 N°7). Most notably, edges became more relevant than the whole area (13 points of Δ AIC when it was 7.9 points of Δ AIC the other way around). Using real coordinates (split) was only marginally better than merging them suggesting that the reality might be between the two contrasted hypotheses represented by these models: the gradient within fields would be neither flat nor as strong as between fields. Finally, estimating the dispersal separately for each site was only marginally better than using the same dispersal parameters for all the sites. The Gaussian kernel still better represented the abundance though the use of the exponential kernel did not change the estimated dispersal characteristics.

### The dispersal is similar in simple and complex landscapes

The best model included an effect of distance from the woods (point structure different from none, Table 5 N°1). The estimation of the mean dispersal distance D_mean_ was similar in the two simple landscapes (1.46 and 1.01 km, Table 5). In the two more complex landscape, D_mean_ tended to be very high at least for the first sampling dates (Table 5). Given that we only accounted for woods within five kilometers would be that the proximity from woodlands did not impact the abundance in complex landscapes, a surprising conclusion when looking at the abundance as a function of the distance from the major woodlands (Fig 2B, Transect 1 and 2).

**Table 5:** Value of fitted dispersal distance global (D_mean_) at each date (D_mean_ (t_n_)) and impact of time on the dispersal (β_dt_) for the best model (N°1) and for the best model without field effect (N°7).

Sites	D_mean_ (t_1_)	D_mean_ (t_2_)	D_mean_ (t_3_)	D_mean_ (t_4_)	D_mean_	β_dt_
Best model with fields (N°1)						
HS	0.6 [0.4,1.2]	0.84 [0.5,1.36]	1.3 [0.8,2.0]	3.0 [1.1,5.4]	1.5 [0.7,2.2]	0.4 [0.2,0.7]
S	0.4 [0.2,0.8]	0.48 [0.3,0.98]	0.8 [0.5,1.4]	2.2 [0.7,5.5]	1.0 [0.5,1.8]	0.7 [0.2,1.3]
C	10.5 [1.2,13.8]	3.62 [1.3,4.93]	2.2 [1.3,3.2]	1.6 [1.0,2.3]	4.5 [1.4,5.7]	-0.2 [-0.2,0.0]
HC	11.7 [1.5,21.1]	6.63 [1.65,13]	4.7 [1.8,9.5]	3.6 [1.8,7.5]	6.6 [1.8,12.7]	-0.1 [-0.1,-0.0]
Best model without fields (N°7)						
HS	0.5 [0.3,0.8]	0.7 [0.5,1.0]	1.0 [0.7,1.5]	2.3 [1.1,13.3]	1.2 [0.8,3.9]	0.5 [0.2,0.8]
S	0.4 [0.2,0.8]	0.5 [0.3,1.0]	0.8 [0.6,1.4]	2.6 [1.1,2.5]	1.1 [0.7,5.4]	0.7 [0.2,1.2]
C	1.2 [0.7,2.3]	1.1 [0.8,1.6]	1.1 [0.8,1.3]	1.0 [0.8,1.5]	1.1 [0.9,1.5]	-0.1 [-0.2,0.1]
HC	1.6 [1.0,3.3]	1.4 [1.02,1.9]	1.2 [1.0,1.5]	1.1 [0.9,1.5]	1.3 [1.0,1.9]	-0.1 [-0.2,0.2]

Model N°1: Time + fields effects, spatial structure of the points within a field, Gaussian kernel, whole area of the woods, dispersal parameters fitted separately for each site.

**Fig 3:**
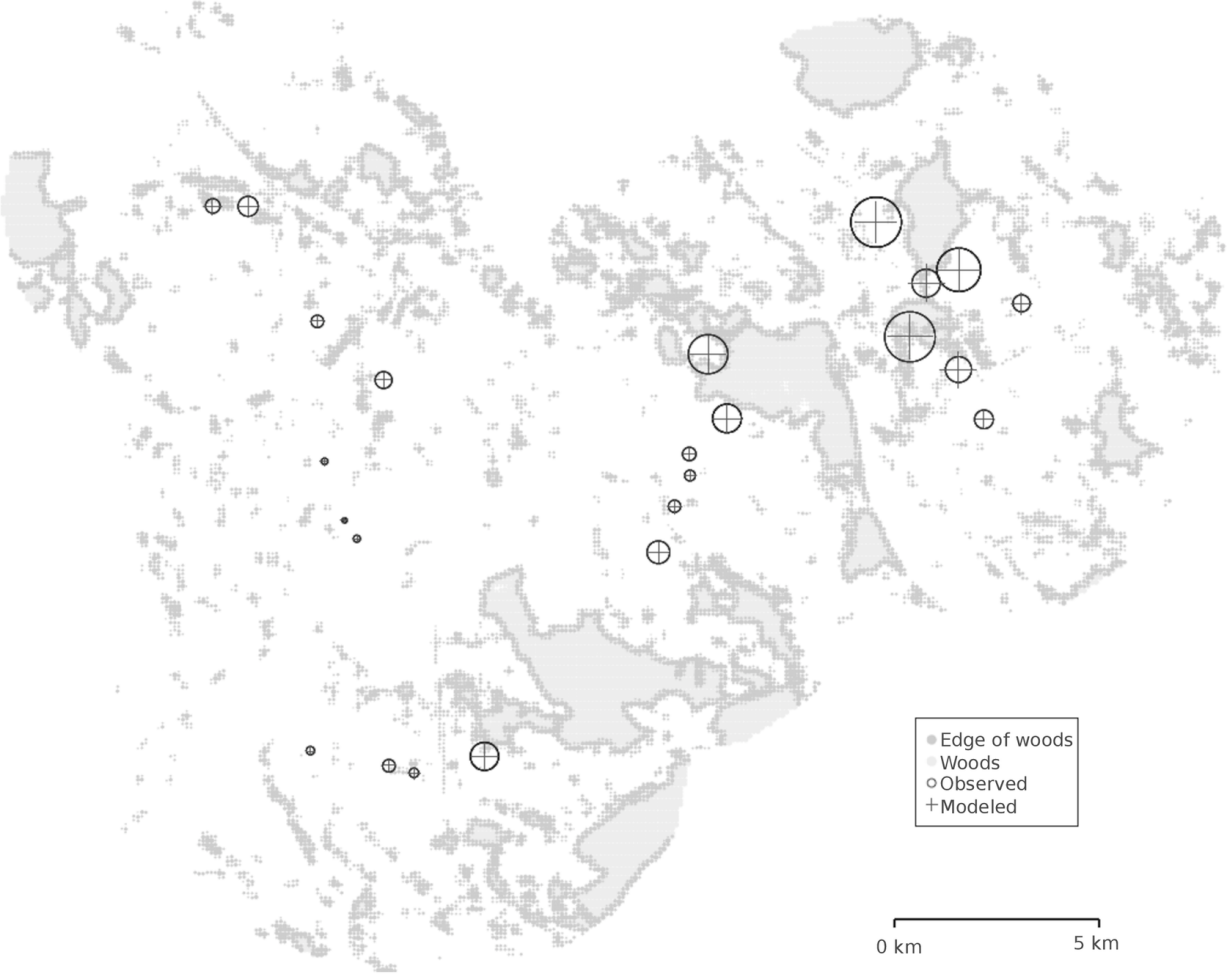
Abundance of pollen beetles observed (circle) and calculated (cross) by the model structured by site and in the context of the surrounding woodlands. Cross and circle sizes were proportional to pollen beetle abundance per 10 plants x 5 points x 4 observations. The surface of the wood area points (edge and core) was proportional to the area of woods in the pixel, with the maximum diameter equal to the pixel width and corresponding to 1ha.

To test if the effect of distance from the woodlands might be hidden by the field effect in more complex landscapes, we looked at the average distances fitted for the best model without field effect (Table 5, N°7). Not only fitting the dispersal separately for the four sites improved the AIC only marginally (Table 5, N°7 vs. 9), but the fitted distances were similar in the four sites and also similar to the distances fitted with the field effect in the simple landscape sites (1.2 km) (Table 5). This suggests that the dispersal was similar in simple and complex landscapes though the field effect tended to hide it (Table 5). When a real impact of the distance to the woods is detected, the average distance traveled by the pollen beetles was close to 1.2 km. The Fig 3 allows to appreciate the correspondence between calculated and observed cumulative abundance per field over the four dates for this model without field effect and the strong relationship between the abundance and the proximity to woodlands (Fig 3). Over the four sites, the dispersal distance also tended to increase with time as the mean β_dt_ was significantly positive for the best models both with field or site effect, when not estimating separately the dispersal parameters per site. The corresponding partial fading out of the gradient from major woods can be visualized in Fig 2B) and the effect (β_dt_ confidence interval not including zero) was particularly clear in simple landscapes (Table 5), suggesting that the pattern might be similar though obfuscated in complex landscapes.

To evaluate if the observed abundance spatial patterns we based our estimations of dispersal on were representative of what can be observed in previous studies [12], [14], [31] we also performed standard buffer based regressions. Specifically we used a generalized linear model (glm) describing the abundance as a simple function of the area of woodlands in buffers of increasing sizes. The best glm model was the model with a buffer radius of 1.8 km.

## Discussion

Many studies have already demonstrated the importance of woodlands in pollen beetle life cycle [14], [15], studied the spatio-temporal patterns of pollen beetle distribution in crops [21] or modeled the immigration of pollen beetle into crops [32] here we explicitly modeled the immigration from woodlands into OSR fields estimating the average dispersal distance and testing additional hypothesis on this dispersal. We estimate the woodlands to be a source of pollen beetle proportionally to their size though the contribution of the center of large woodlands remains unclear. With the weeks, the gradient of pollen beetles from woodlands tended to fade out which translate in higher dispersal distances estimated. The pollen beetle likely dispersed with a similar mean dispersal distance in simple or complex landscapes. However, in complex landscapes, the estimation of the dispersal seems to have been obfuscated by other non-measured factors. The variability of the abundance between fields was largely explained by the dispersal from neighboring woods but there was as considerable remaining variability between dates, sites and, to a lesser extent, between fields.

Dispersal from the woodlands continued in OSR fields, but within the fields we observed a high variability of the abundance that is not explained by the distance to surrounding woodlands. Models accounting or not for the exact position of the sampling point in the field had similar results suggesting the gradient within fields was neither flat nor as strong as between fields. The high variability of the abundance within fields suggests that within fields the abundance is strongly influenced by other factors here not assessed. This would be nevertheless surprising given that the aggregation of pollen beetles in fields has been documented [33]. The OSR plants might have different attractiveness as pollen beetles abundance is correlated with the OSR growth stage [24]. Pollen beetles also tend to aggregate on blossomed OSR flowers, and flowering is not homogenous in the field [13]. Other studies found more pollen beetles at the field edges than at the center [20], [32] (six times more pollen beetles at the field edges). We might not see such a strong effect because OSR fields in our study were smaller: mean area of 25 ha, compared to 45 ha for [20], leaving less distance for the gradient to express itself. Alternatively, as other studies suggest that pollen beetle fly upwind into crops [16], transects might observe gradients of varying strength depending on the existence of a strongly prevailing wind and on the alignment of the transect to such prevailing wind.

Our estimation of the effect of the distance in the different sites suggests that the dispersal is similar in complex and simple landscapes, nevertheless the effect of the woodlands on the abundance can be obfuscated in complex landscapes, but only at early dates. Difficulties to observe a statistical relationship between woodland areas and pollen beetle though unusual is not unprecedented [16]. In our particular case this effect is only hidden in complex landscapes and at early dates, a possible explanation could be that complex sites present more small overwintering sites such as hedgerows or grasslands [11]. The emergence of the pollen beetle happens when a sum of degree per day is attained [2], [16]. As non-wooded areas and even small wooded areas such as hedgerows warm up faster [34], pollen beetles overwintering into them might emerge faster and hence be early colonizer of OSR crops. In our complex landscape sites that had more grassland and hedgerows in the landscape, these potentially early colonizer could quickly spread all over the site blurring the nascent gradient of abundance from the woodlands. One could think that the semi-natural habitats such as hedgerows also had plentiful flowers and affected the dispersal as they might serve as relay providing resources to pollen beetle leaving their overwintering habitat, nevertheless to blur the signal only for the first dates of observations as we observed, such relay would have to 1) increase the dispersal a lot and 2) increase it only for the first pollen beetle emerging, neither proposition being sustained by published data to our knowledge, we hence favor our first hypothesis.

We find an important time effect with a maximum abundance of pollen beetle at the third date. An overall bell shape is expected as the wave of pollen beetle getting out of overwintering habitats swashes the neighboring habitats [12], [16] in addition crop attractiveness is maximal at blossom favoring such maximum of abundance [24]. Finally we observe very few pollen beetle on the second date that corresponds to a rainy and windy day limiting the observability of the pollen beetle [16].

As pollen beetles oviposit in buds, the growth stage of the OSR is a major parameter for the damage pollen beetles could cause. As the growth stage is linked to the date, early arriving pollen beetles are more likely to find non blossomed flowers and damage it [6], [13]. We found, at least in the simple landscapes, a significant effect of time on the dispersal range, confirming our hypothesis that with time they tend to move further away from woodlands. Alternatively, the dispersal might be very quick but highly density dependent: as the later dates are the one with more pollen beetles, they might go further when they are more numerous to emerge. Finally, the flight dispersal could also be temperature dependent; with warmer temperatures towards the end of the season pollen beetles are more likely to fly and could fly further [35]. In any case, the fields closer to the woods are then not only attacked by more pollen beetles but also when the crops are most sensitive to the attacks [36]. This could extend to OSR fields close to hedgerows or even grasslands if as suggested above hedgerows and grasslands are a significant source of pollen beetle early in the spring.

Overall, our study suggests a mean dispersal distance of 1.2 km, confirming a posteriori that the mapping of the woodlands within five kilometers of the OSR was enough. With both the Gaussian and the exponential kernels, we found a mean dispersal distance of 1.2 km, even if the exponential kernel had a fatter tail. In any case, these results suggest that dispersal movements are local [6] and that high abundance should rarely be found at ten kilometers from over-wintering sites. This estimation is largely in agreement with some estimations of pollen beetles dispersal based on mark-release-recaptures experiments [18] but differs from other estimates suggesting a dispersal distance of ten kilometers in two days [17]. One can note the very artificial conditions of release of these pollen beetles in mark-release-recapture. The feeding status and stress level of the insects as well as the date of release might have a strong impact on the dispersal; at the latest date, the average dispersal of 2.6 km we estimate in simple landscapes would be for example much more compatible with occasional flight over 10 km. Other statistical studies associated the abundance of pollen beetles with the area of woodlands within 1.5 to 2 kilometers of the sampling point [12], [14], [31]. This is in complete agreement with the maximum of likelihood we find in our data for models based on buffers of 1.8 km radius despite the fact that we observed much lower pollen beetle abundance per plant, suggesting that our estimate of dispersal is relevant for other times and places.

We found that the Gaussian kernel is slightly better than the exponential kernel and the conclusions are similar using one or the other kernel. The Gaussian kernel corresponds to a random dispersal while the exponential kernel corresponds to a centrifuge, dispersal from woodlands, with random stopping time [26]. The dispersal of the pollen beetles might be closer to the former. This is in agreement with [17], suggesting that *B*. *aeneus* fly in all the directions, with no effect of wind or topography. Nevertheless, upwind anemotaxis toward oilseed rape has been reported for overwintered *B*. *aeneus* [2], [16], [37]. In regions with a strong prevailing wind, using such symmetric kernels might not be adequate and more generally estimating a mean dispersal might not be relevant without considering the direction of the wind. Nevertheless the pollen beetle tend to not fly in strong winds, limiting the impact of strong dominant winds and in our case, in eastern Normandy, there is no clearly prevailing wind [38]. The use of pesticides could also potentially have a strong impact on the abundance and dispersal of the pollen beetle. In particular, a generalized use of insecticide on OSR before blossom followed by a diminution of the insecticide use after blossom could induce both the increase of the observed population of pollen beetle with time and perturbation of the dispersal gradient early on. One could also argue that the OSR is more treated where pollen beetles are more abundant. This would tend to erase the gradient we observe from woodlands leading to higher estimated dispersal distance. It could be an explanation of the limited gradient we observed in complex landscapes. Nevertheless, overall, we observe a very clear gradient and the dispersal distance we estimate tend to be small compared to previous estimations by mark-release-recaptures. In any cases, our estimates are relevant for landscapes with similar insecticide pressure and conclusions on the dispersal of pollen beetle in landscapes fully exempt from insecticides might be significantly different. Abundance and potentially dispersal might also be affected by other OSR management practices such as cultivar used [8] or sowing dates as it impacts the flowering dates [39]. We do not expect those to be spatially correlated to the presence of woodlands limiting the impact on our estimates of the dispersal distance from woodlands to OSR. In general the overall stability of the dispersal distance estimates over the different sites at least for the later dates suggests a limited impact of the disparity of these practices in the landscape on the dispersal distance.

Depending on the model the source of pollen beetle to consider to best explain the data is the 100 m wide circumference of the woodlands or the whole woodlands. This surprising inversion might be due to a confusion between the site effect and the presence of woodlands large enough to have a core of a significant size within the 100 m circumference (Fig 3). It might also be linked to a higher semi-natural habitat density in the complex landscapes. In any case, there is no strong support for having less pollen beetles in the center of the woodlands. This is coherent with previous work finding that the pollen beetles overwinter at similar densities in edge under 10 m and at 50 to 100 m into woodlands [11]. The variability of *B*. *aeneus* capture related to the date (strong diminution of pollen beetles abundance the first of April) also confirms the impact of the weather on the sampling which was realized under light rain. This also confirms that rains can limit pollen beetles damage on OSR [17], [40].

We focused here on the dispersal distance of the pollen beetle from woodlands to OSR fields. Understanding how other land use might influence the dispersal could be key to manage the pollen beetle invasions. In particular, though the kernel based formalism we use does not imply that the dispersal is direct from the woodlands to the OSR fields, it might be beneficial to account explicitly for putative relay such as grasslands, hedgerows or wild flowers on the road borders [2] Such refinements of our model could help explain the differences observed between the sampled sites, currently aggregated in our catch all categorical term γ_*g*_. It would imply to better cartography the land uses but would also very significantly increase the computation time for the simulations. The abundance of pollen beetles overwintering in woodland could also vary according to the presence of previous OSR fields close to overwintering sites [16]. The surface of the OSR of the year N-1 at proximity of the woodlands could influence the load in these woodlands and participate to the site effect. Furthermore, woodlands and hedgerows characteristics such as moisture, exposition or litter thickness are important for pollen beetle overwintering [11].

Finally, natural enemies such as predators [41], [42], fungus [43], or parasitoids [42] may limit the growth of pollen beetle population. It could be argued that the observed patterns of abundance might be shaped by the predation of the pollen beetles by generalist predators such as birds. This is unlikely though as predation, as well as parasitism is expected to appear mostly around semi-natural landscapes and woods and hedgerows in particular [12], in clear opposition with the higher abundance observed here close to woods and in complex landscape sites. A possible control by natural enemies more intense around woodlands does not seem to counter the strong direct impact of these features on the life cycle of the pollen beetle.

We show that the average dispersal of the pollen beetle is over 1.2 km, inducing a very strong dependence of their abundance to the abundance of nearby woodlands or potentially other over-wintering sites [11]. Within field variability in pollen beetle abundance was high. Finding the right attractors allowing to use trap crops as a within field management strategy [7], [9], possibly with earlier flowering [44] or adequate volatile compounds [8] might be efficient though difficult. Complex landscape do not seem to have a clear impact on the dispersal of the pollen beetle from the woodland. As this dispersal is not stopped either by intermediary OSR fields using “trap fields” nearby woodlands to limit pollen beetles dispersal does not seem relevant. Nevertheless, OSR fields close to woodlands are both more infested and to some extent infested earlier than OSR fields far from woodlands. To reduce the risk of OSR field to be colonized by pollen beetle when they are close to woodlands, plantation of early OSR flowering varieties may be recommended.

## Supporting information

S1 Table Models obtained constraining in turn each parameter modality. Factor type: type of factor. Origin: whole wood area or only 100 m wide area bordering woodland area. Same field point structure: split = with real coordinates of points. Same field point structure = all points of a field with coordinates of barycenter, none = no coordinates. Kernel = Exponential (exp.) or Gaussian (Gaus.) dispersal kernels. Site dependent dispersal: with or without interaction between site and distance. Mean β_dt_: mean effet of time on the dispersal distance. Mean D_mean_: mean distance of dispersal from woodlands.
(XLSX)

S2 Table Abundance data used for this paper. Field: field sampled, Site: one of the four study sites. Date: sampling date, Pollen beetles Number: Number of pollen beetles for ten plants. X and y: coordinates in metric unit.
(XLSX)
